# Behavioral and cognitive data in mice with different tryptophan-metabolizing enzymes knocked out

**DOI:** 10.1016/j.dib.2016.08.071

**Published:** 2016-09-06

**Authors:** Lay Khoon Too, Kong M. Li, Cacang Suarna, Ghassan J. Maghzal, Roland Stocker, Iain S. McGregor, Nicholas H. Hunt

**Affiliations:** a Molecular Immunopathology Unit, Bosch Institute and School of Medical Sciences, University of Sydney, Sydney, New South Wales 2006, Australia; b Discipline of Pharmacology, Sydney Medical School, University of Sydney, Sydney, New South Wales 2006, Australia; c Vascular Biology Division, Victor Chang Cardiac Research Institute, Darlinghurst, 2010, Australia and School of Medical Sciences, Faculty of Medicine, University of New South Wales, NSW 2052, Australia; d School of Psychology, Faculty of Science, University of Sydney, Sydney, New South Wales 2006, Australia

## Abstract

This article demonstrates behavioral changes in mice in response to free adaptation and drinking session adaptation modules implemented in their social home environment, the IntelliCage. These data complement the study “Deletion of *TDO2*, *IDO-1* and *IDO-2* differentially affects mouse behavior and cognitive function” (Too LK, Li KM, Suarna C, Maghzal GJ, Stocker R, McGregor IS, et al., 2016) [1]. Prior to programmed drinking sessions, all mice were exposed to a home cage adaptation module during which there was no time limit on water access – the free adaptation module. The exploratory behaviors are here expressed as percentages of visits with nosepokes and of visits with licks. The measurements by percentage of exploratory activity showed minimal genotype effects. The number of nosepokes or licks per corner visit also was compared between WT and gene knockout (GKO) *IDO1* mice, WT and GKO *IDO2* mice and WT and GKO *TDO2* mice and demonstrated unremarkable behavioral changes during the free adaptation module. Analysis of drinking session adaptation behavior showed no genotype effect between WT and GKO of *IDO1*, *IDO2* or *TDO2* background. Notwithstanding the absence of genotype differences, each IDO1, IDO2 or TDO2 animal group displayed a specific pattern of adaptation to the drinking session modules. Furthermore, IDO1 GKO mice showed a more rapid recovery of lick frequency to the baseline level compared to the WT equivalents in a simple patrolling task during the first complete testing cycle (R1). TDO2 GKO mice on the other hand did not differ from their WT equivalents in terms of lick frequency over the three test days of complex patrolling and discrimination reversal tasks. Lastly, IDO2 GKO mice reduced their visits to the permanently non-rewarding reference corners by the same degree as did the WT mice.

**Specifications Table**

**Table:** 

Subject area	*Psychology*
More specific subject area	*Mouse behavior and cognition*
Type of data	*Figures*
How data were acquired	IntelliCage™ (New Behavior AG, Zurich, Switzerland; http://www.newbehavior.com)
Data format	*Analyzed, graphed*
Experimental factors	*Genetic engineering* to eliminate one of the three tryptophan-metabolizing enzymes – IDO1, IDO2 and TDO2 – in mice.
Experimental features	*Behavioral analysis of mice subjected to adaptation and learning modules in the IntelliCage*
Data source location	*The University of Sydney, New South Wales, Australia*
Data accessibility	*Data are with this article*

**Value of the data**

• The experimental approach and data allow comparative evaluation of baseline nosepoking and licking behaviors as percentages of total corner visit frequency, and nosepoke or lick frequency in every visit, of mice with or without a functional *IDO1*, *IDO2* or *TDO2* gene.
• The data are valuable for understanding behavioral adaptation to the introduction of fixed drinking sessions (i.e. availability of water as a reward) by mice deficient in the *IDO1*, *IDO2* or *TDO2* genes.
• The data make clear that the genetic background of different mice may influence adaptative behavior to the drinking sessions more than the gene modifications tested, emphasizing that studies with GKO mice must employ WT mice of the identical strain.
• It is shown that mice deficient in IDO1, IDO2 or TDO2 enzymes exhibit different cognitive changes compared to their WT equivalents, which is relevant to understanding the role of the kynurenine pathway of tryptophan metabolism in behavioral adaptation.

## 1 Data

**Fig. 1:**
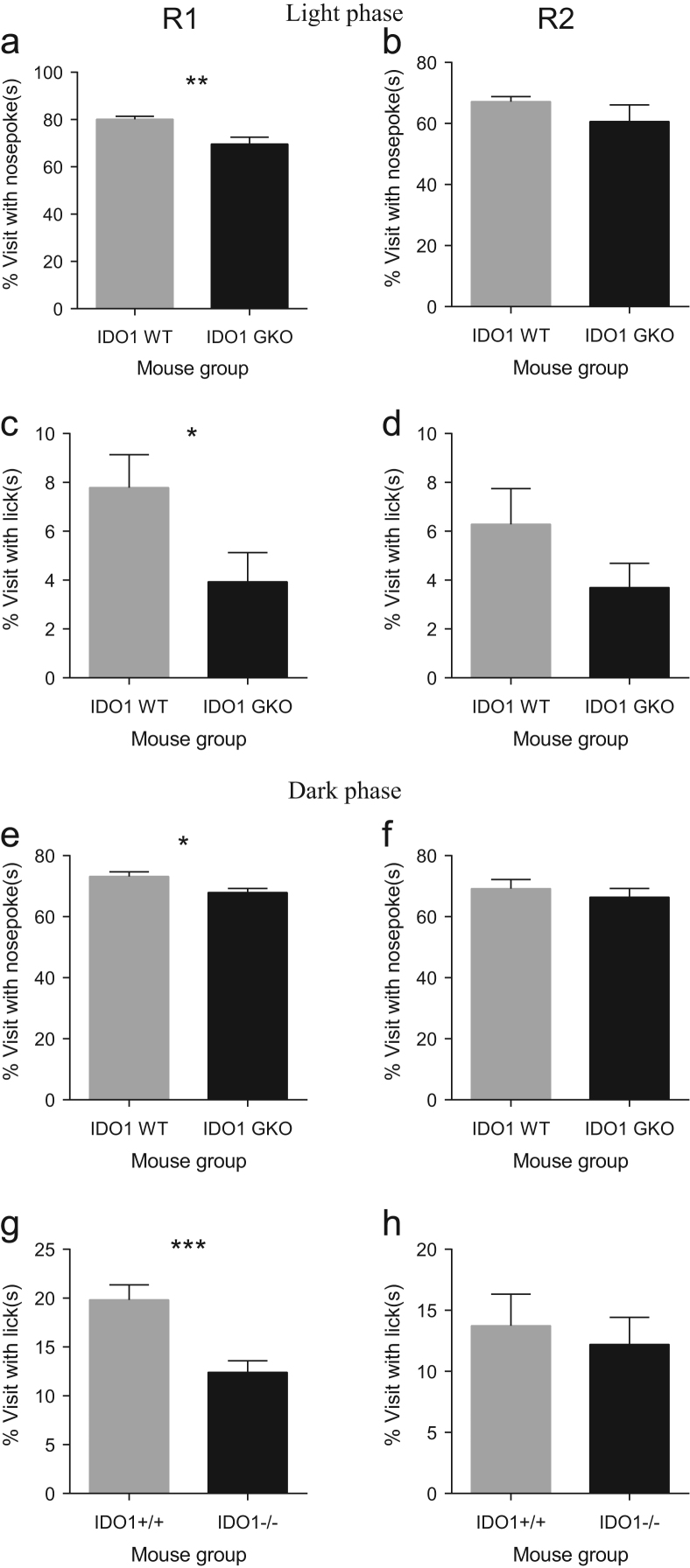
IDO1 deficiency causes reduction in percent visits with nosepokes and visits with licks in mice after 5 h of free adaptation in R1 and R2 during the light and dark phases. Deficiency of IDO1 resulted in a significant decrease in percent visits with nosepokes in R1 after 5 h of exploration in the light (**a**) and dark (**e**) phase of free adaptation, but not in R2 (**b, f**). Likewise, *IDO1* GKO mice made proportionally less visits with licks in R1 compared to their WT counterparts during the light (**c**) and dark (**g**) phase. A significant genotype effect was not observed in R2 in terms of percent visits with licks (**d, h**). Data are mean±SEM. *p<0.05, **p<0.01, ***p<0.001, unpaired *t*-test with Welch׳s correction in the event of unequal variances. Total *n*=16 mice per WT or GKO group.

**Fig. 2:**
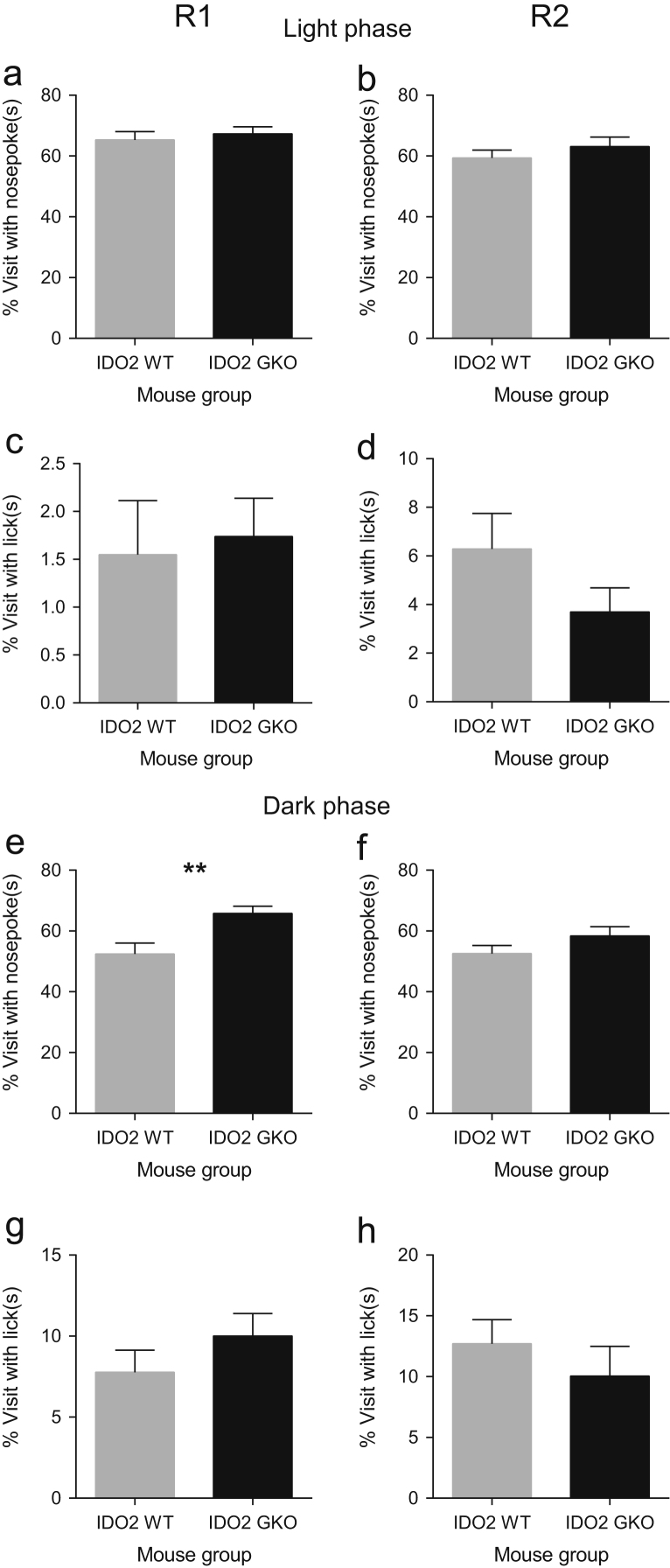
IDO2 deficiency is associated with minimal change in exploratory activity by percentage of total visit frequency after 5 h of free adaptation during the dark phase of R1. Percent visits with nosepokes after 5 h of exploration in the light phase during R1 (**a**) and R2 (**b**) were not significantly different between WT and GKO mice. There was also no significant genotype effect on percent visits with licks after 5 h of exploration in light phase during R1 (**c**) and R2 (**d**). Deficiency of IDO2 resulted in a significant increase in percent visits with nosepokes after 5 h of nocturnal exploration in R1 (**e**), but not in R2 (**f**). A significant genotype effect was however not observed in both R1 (**g**) and R2 (**h**) in terms of percent visits with licks during the dark phase (**d, h**). Data are mean±SEM. **p<0.01, unpaired *t*-test with Welch׳s correction in the event of unequal variances. Total *n*=16 mice per WT or GKO group.

**Fig. 3:**
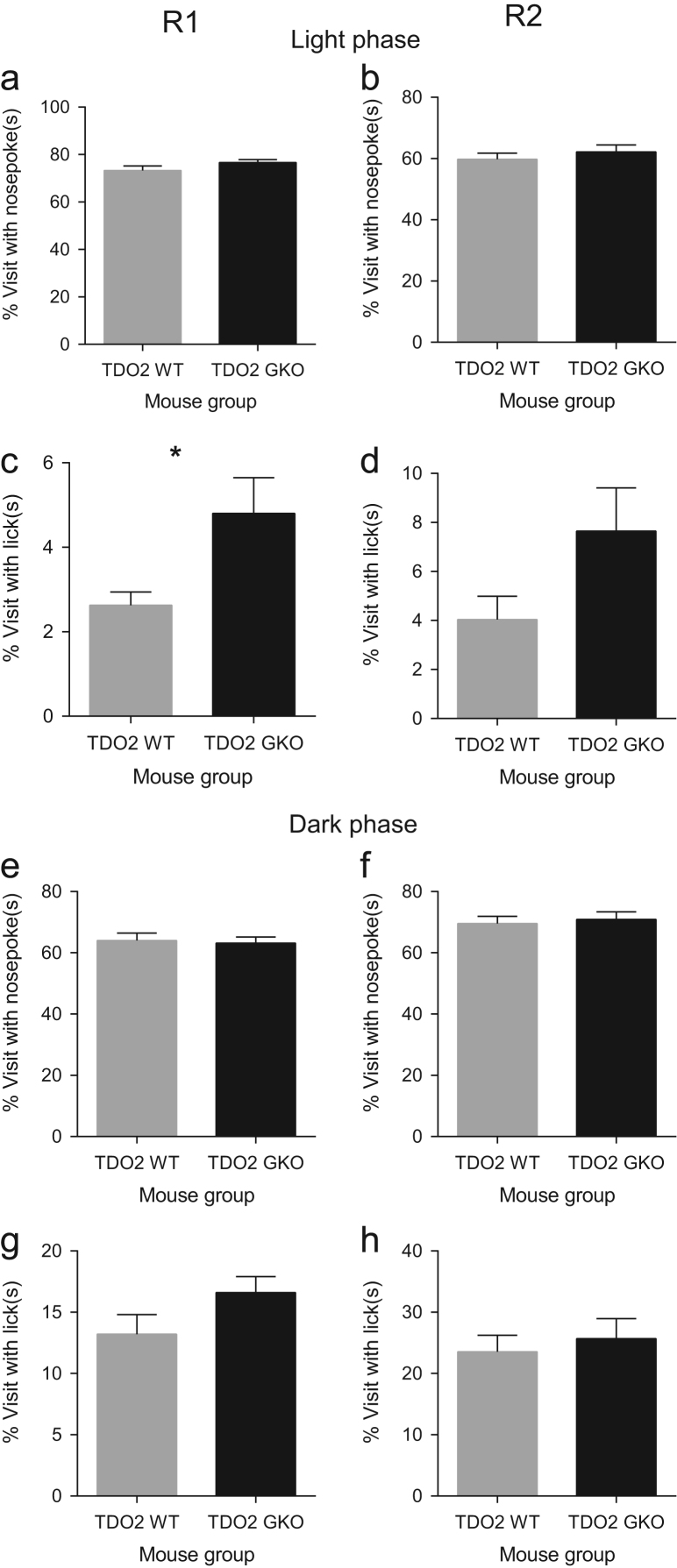
TDO2 deficiency is associated with minimal change in exploratory activity as measured by percentage of total visit frequency after 5 h of free adaptation during the light phase of R1. Percent visits with nosepokes after 5 h of exploration in the light phase during R1 (**a**) and R2 (**b**) was not significantly different between WT and GKO mice. A significant genotype effect on percent visits with licks after 5 h of diurnal exploration was observed in R1 (**c**), but not in R2 (**d**). Deficiency of TDO2 did not affect percent visits with nosepokes after 5 h of nocturnal exploration in R1 (**e**) and R2 (**f**). A significant genotype effect also was not observed in either R1 (**g**) or R2 (**h**) in terms of percent visits with licks during the dark phase (**d, h**). Data are mean±SEM. *p<0.05, unpaired *t*-test with Welch׳s correction in the event of unequal variances. Total *n*=18 WT and *n*=17 GKO mice.

**Fig. 4:**
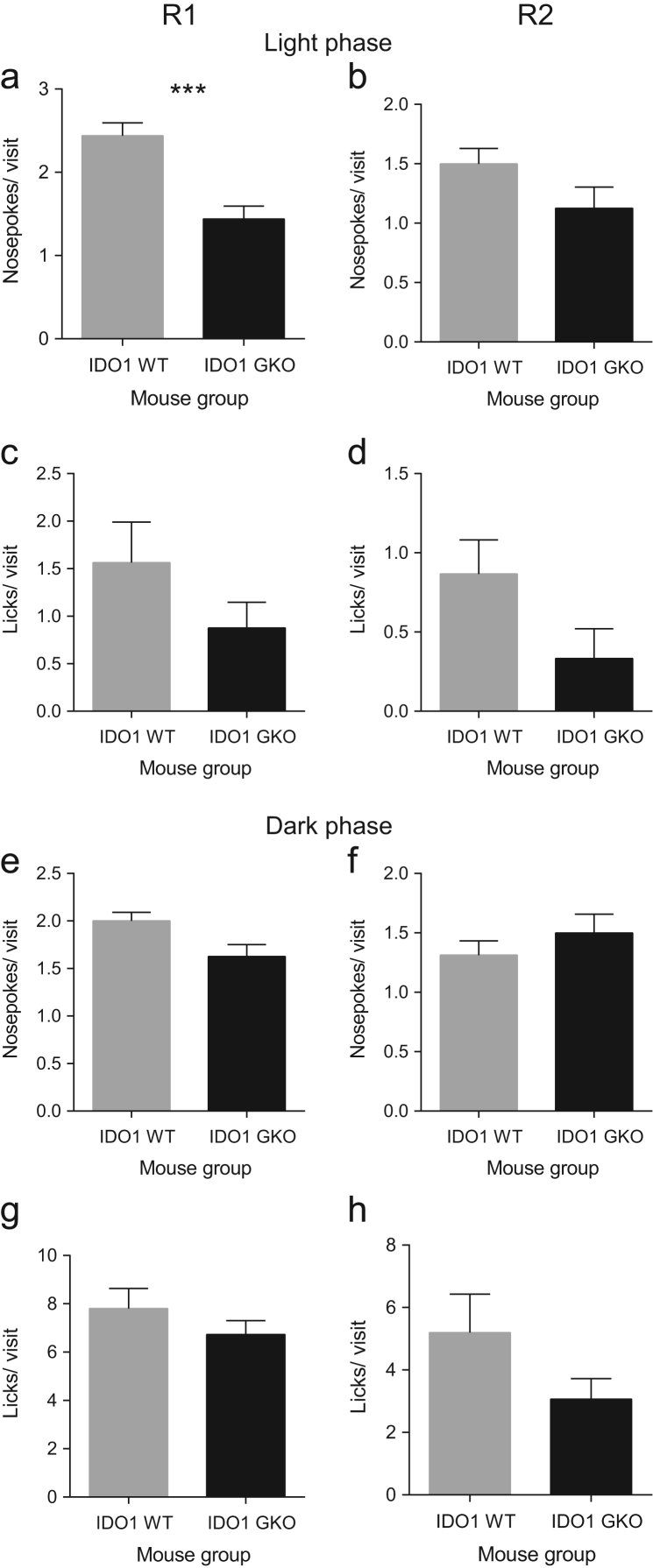
IDO1 deficiency is associated with minimal change in average number of nosepokes in every visit after 5 h of free adaptation. The average number of nosepokes per diurnal visit was significantly lower in GKO than WT mice in R1 (**a**) but not in R2 (**b**). The average number of licks in every diurnal visit was however not significantly different between GKO and WT mice in R1 (**c**) and R2 (**d**). In the dark phase, IDO1 deficiency did not alter the average number of nosepokes made by mice in every visit in both R1 (**e**) and R2 (**f**). Likewise, there was no significant genotype effect on the average number of licks per visits made by mice during the dark phase of R1 (**g**) and R2 (**h**). Percent visits with nosepokes after 5 h of exploration in the light phase during R1 (**a**) and R2 (**b**) was not significantly different between WT and GKO mice. A significant genotype effect on percent visits with licks after 5 h of diurnal exploration was observed in R1 (**c**), but not in R2 (**d**). Deficiency of TDO2 did not affect percent visits with nosepokes after 5 h of nocturnal exploration in R1 (**e**) and R2 (**f**). Data are mean±SEM. ***p<0.001, Mann–Whitney test. Total *n*=16 WT and *n*=16 GKO mice.

**Fig. 5:**
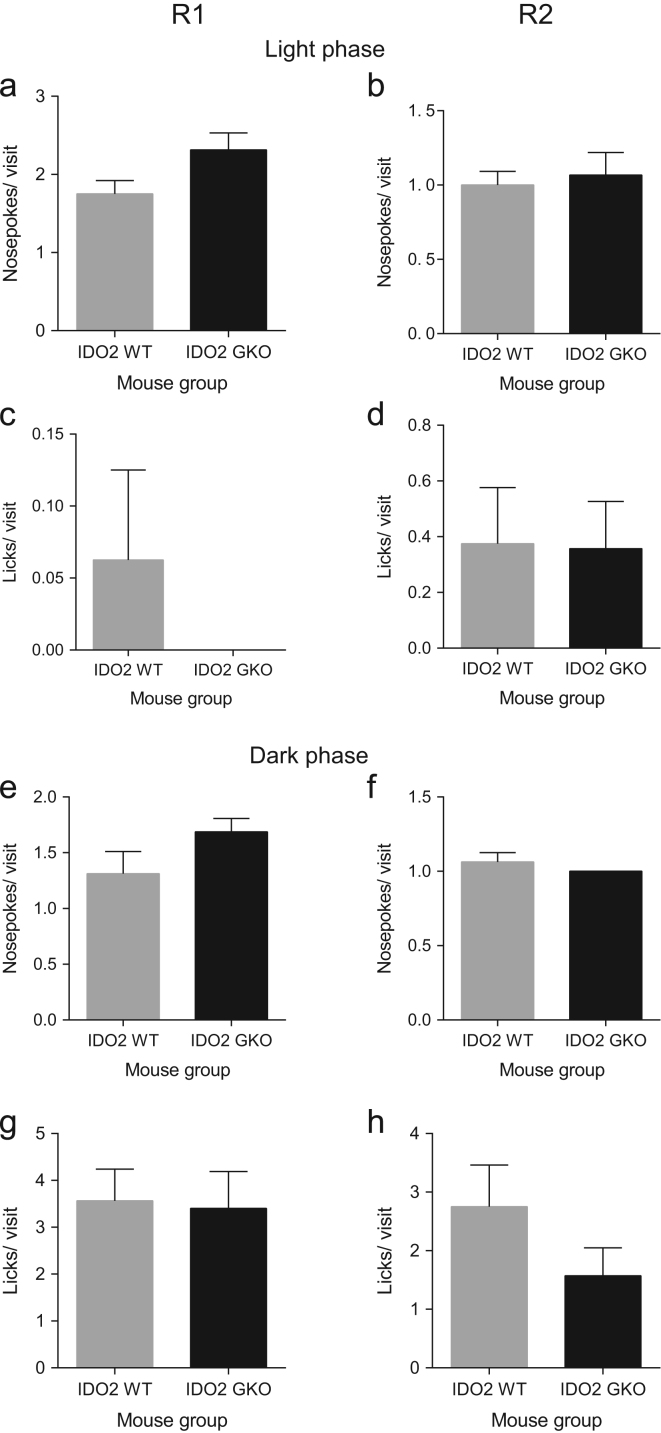
IDO2 deficiency does not change the average number of nosepokes or licks in every visit made by mice after 5 h of exploration in the free adaptation period. The average number of nosepokes or licks per diurnal visit made by WT mice was not significantly different from GKO mice in R1 and R2 (**a–d**). During the dark phase, a significant genotype effect comparing WT and GKO mice also was absent in both R1 and R2 in terms of the average number of nosepokes or licks per nocturnal visit (**e–g**). Data are mean±SEM. Mann–Whitney test. Total *n*=16 WT and *n*=16 GKO mice.

**Fig. 6:**
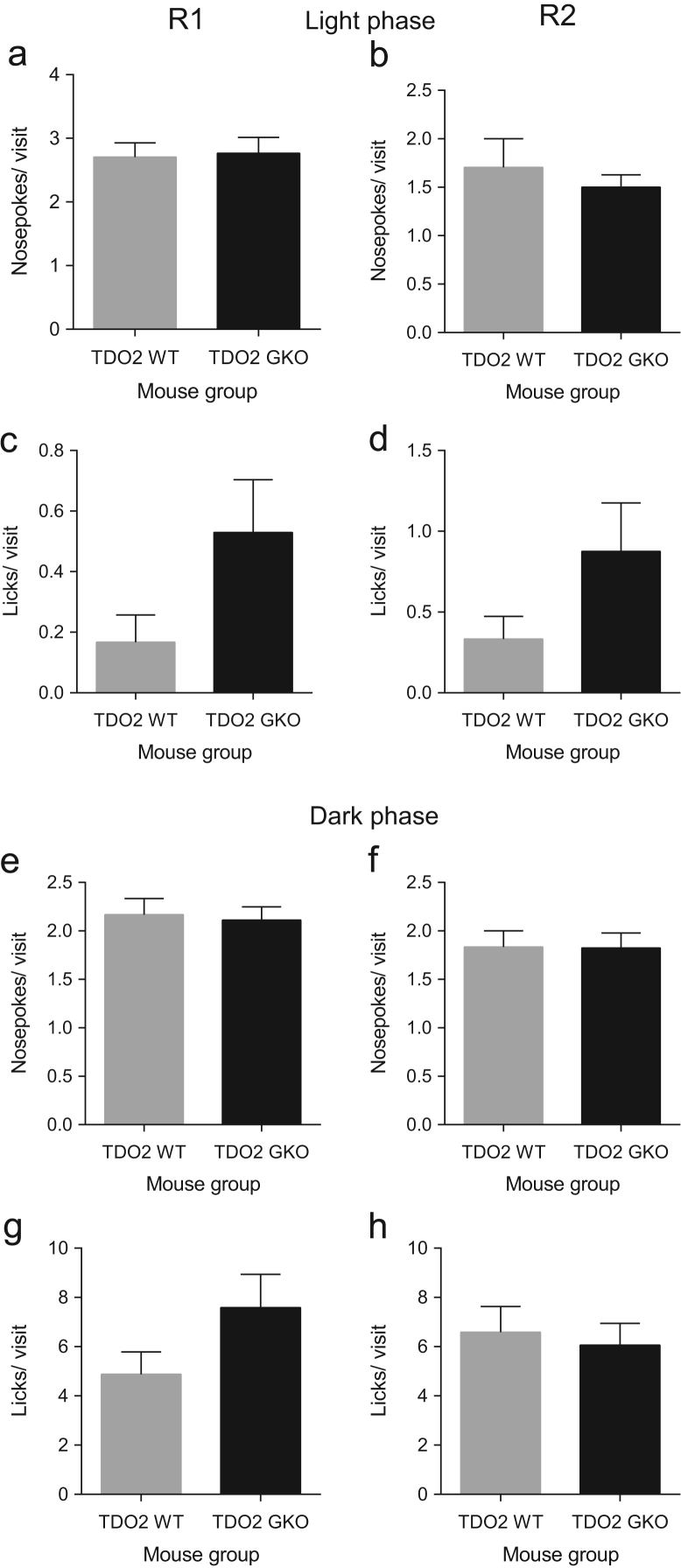
TDO2 deficiency does not affect the average number of nosepokes or licks in every visit made by mice after 5 h of exploration in the free adaptation period. The average number of nosepokes or licks per diurnal visit made by WT mice was not significantly different from GKO mice in R1 and R2 (**a–d**). During the dark phase, a significant genotype effect comparing WT and GKO mice also was absent in both R1 and R2 in terms of the average number of nosepokes or licks per nocturnal visit (**e–g**). Data are mean±SEM. Mann–Whitney test. Total *n*=18 WT and *n*=17 GKO mice.

**Fig. 7:**
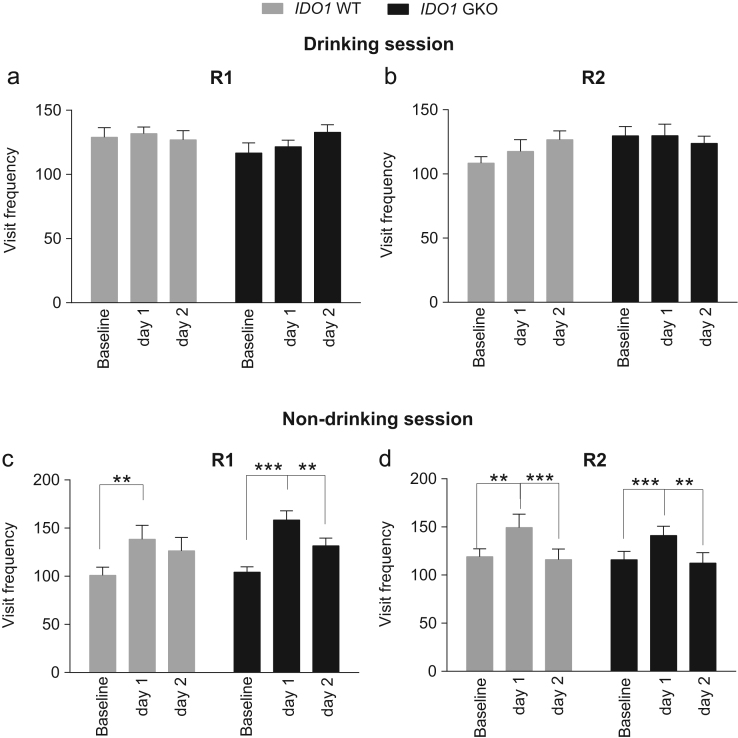
Corner visit frequency of ***IDO1*** WT and GKO mice during drinking and non-drinking sessions in R1 and R2. In comparison to the day before drinking sessions were executed (“baseline”), in both R1 (**a**) and R2 (**b**) the IDO1 WT and GKO mice maintained the same level of corner visit frequency throughout the drinking sessions. However, they significantly increased their corner visits during non-drinking sessions on the first day, then decreased them on the next day, in R1 (**c**) and R2 (**d**), implying that they had become aware that water rewards were not available in some sessions. The visit frequency of test day 1 or 2 was compared to that on the previous day by repeated contrast. The data (mean±SEM) showed no significant genotype effect at each level of repeated contrast. *p<0.05, **p<0.01, ***p<0.001, repeated contrast. Total *n*=16 mice per WT or GKO group.

**Fig. 8:**
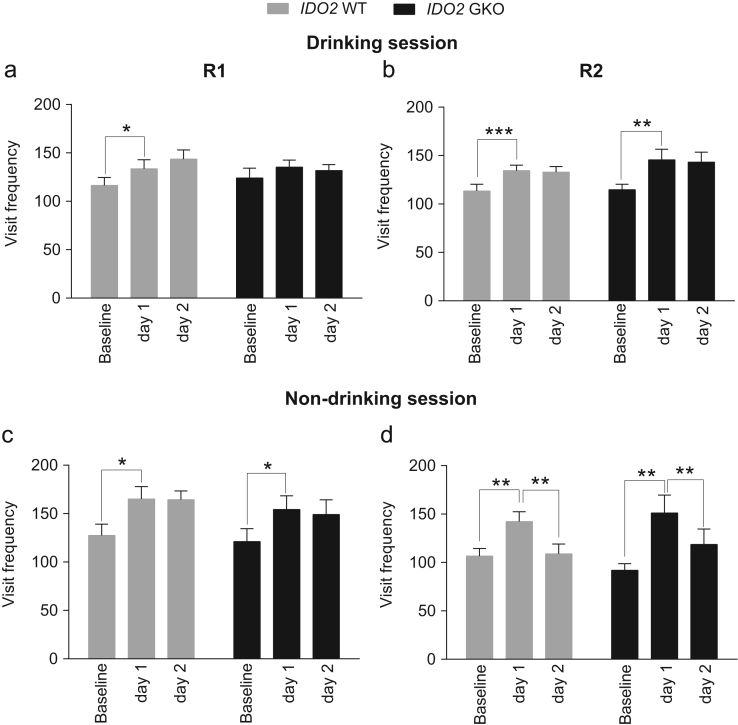
Corner visit frequency of IDO2 WT and GKO mice during drinking and non-drinking sessions in R1 and R2. During the drinking sessions, IDO2 WT mice significantly increased their corner visits in the first day while retaining the same level of corner visits for the next day in both R1 (**a**) and R2 (**b**). No significant genotype effect was observed between WT and GKO mice. (**c**) During non-drinking sessions, both WT and GKO mice significantly increased corner visits only on the first day in R1. (**d**) However, in R2 they significantly reduced visit activity on the next day, suggesting learned behavior. The visit frequency of test day 1 or 2 was compared to that on the previous day by repeated contrast. *p<0.05, **p<0.01, ***p<0.001, repeated contrast. The data (mean±SEM) showed no significant genotype effect at each level of repeated contrast. Total *n*=16 WT and *n*=15 GKO mice.

**Fig. 9:**
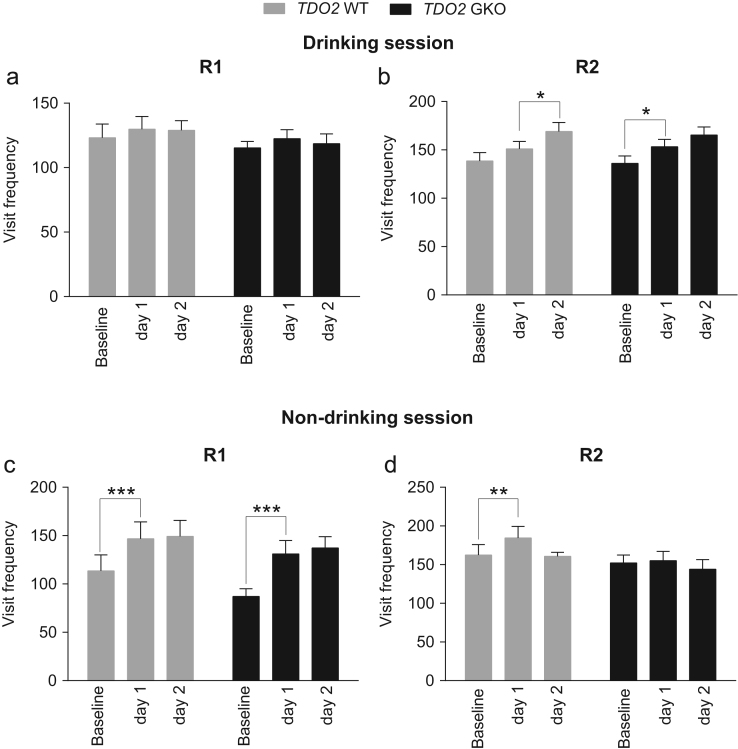
Corner visit frequency of TDO2 WT and GKO mice during drinking and non-drinking sessions in R1 and R2. (**a**) Over the 2-day drinking session, in R1, both *TDO2* WT and GKO mice retained their corner visit behavior. (**b**) By contrast, in R2, WT and GKO mice increased corner visit frequency in the 2-day drinking session. There was no significant genotype effect relating to this. (**c**) Both WT and GKO mice increased corner visits on the first execution of non-drinking session in R1. (**d**) The increase in corner visits during the non-drinking session on the first test day was observed only in WT mice, but there was no significant genotype effect at each level of repeated contrast. Data showed mean±SEM. The visit frequency of test day 1 or 2 was compared to that on the previous day by repeated contrast. *p<0.05, **p<0.01, ***p<0.001, repeated contrast. Total *n*=18 WT and *n*=17 GKO mice.

**Fig. 10:**
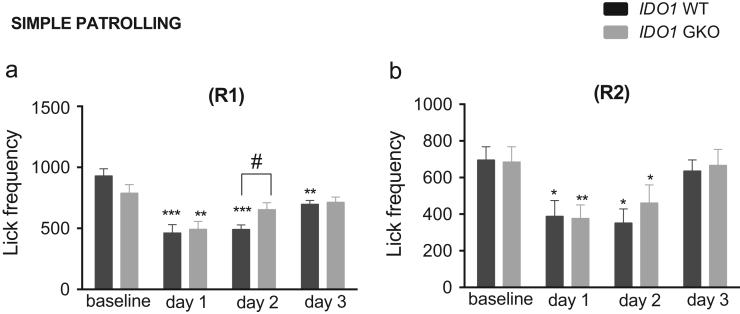
IDO1 genotype effect on lick frequency when mice were tested in simple patrolling. The frequency values were reported in R1 **(a)** and R2 **(b)**. *p<0.05, **p<0.01, ***p<0.001, simple contrast with reference category set at baseline. #*p*<0.05, genotype x test day simple contrast. Total *n*=16 mice per WT or GKO group.

**Fig. 11:**
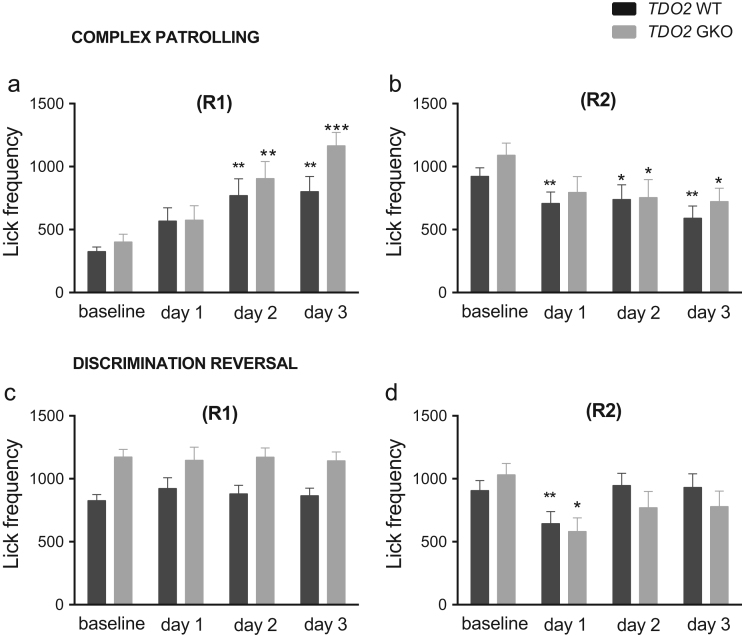
Frequency of licks when ***TDO2*** GKO and WT mice were tested in complex patrolling and discrimination reversal tasks. The frequency values were reported in R1 and R2 of complex patrolling (**a**, R1; **b**, R2) and discrimination reversal task (**c**, R1; **d** R2). *p<0.05, **p<0.01, ***p<0.001, simple contrast with reference category set at baseline. Total *n*=18 WT and *n*=17 GKO mice.

**Fig. 12:**
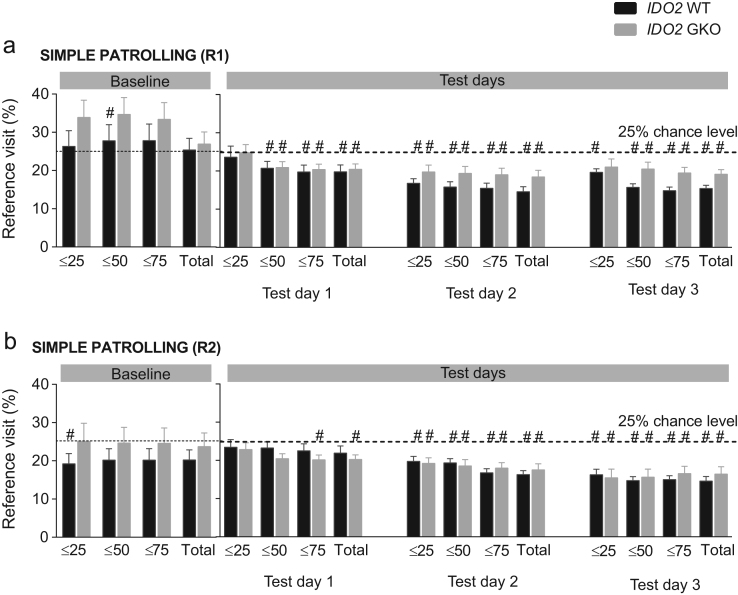
IDO2 genotype effect on reference memory when mice were tested in simple patrolling. The percentage of visits to the reference corner was measured in R1 **(a)** and R2 **(b)**. A significant genotype effect was absent. #*p*<0.05, genotype x test day simple contrast. Total *n*=16 WT and *n*=15 GKO mice.

The current data show distinct behaviors of WT and GKO mice deficient in the *IDO1* (Fig. 1, Fig. 4, Fig. 7), *IDO2* (Fig. 2, Fig. 5, Fig. 8) or *TDO2* (Fig. 3, Fig. 6, Fig. 9) genes when they were subjected to a drinking session test module in the IntelliCage system. In addition, the lick frequency of mice when subjected to cognitive tests was compared between the WT and GKO of *IDO1* mice (Fig. 10) as well as between the WT and GKO of *TDO2* (Fig. 11) mice to evaluate the reward-driven behavior that was potentially associated with the altered cognitive performance. Moreover, the percentage of reference visits made by WT and GKO IDO2 mice in simple patrolling was compared (Fig. 12), to provide additional data to support the observed genotype effect on this parameter in the complex patrolling task (see Fig. 5 in Ref [1]).

## 2 Experimental design, materials and methods

The data involve the same set of animals and methods as described [1]. Mice were initially trained with the free adaptation and nosepoke adaptation modules prior to the drinking session adaptation. The three adaptation modules and the cognitive function test modules have been described in detail previously [1].
